# Natural disaster preparedness in a multi-hazard environment: Characterizing the sociodemographic profile of those better (worse) prepared

**DOI:** 10.1371/journal.pone.0214249

**Published:** 2019-04-24

**Authors:** Nicolás C. Bronfman, Pamela C. Cisternas, Paula B. Repetto, Javiera V. Castañeda

**Affiliations:** 1 Engineering Sciences Department, Universidad Andres Bello, Santiago, Chile; 2 National Research Center for Integrated Natural Disaster Management CONICYT/FONDAP/15110017, Santiago, Chile; 3 Industrial and Systems Engineering Department, Pontificia Universidad Catolica de Chile, Santiago, Chile; 4 Department of Psychology, Pontificia Universidad Catolica de Chile, Santiago, Chile; Bielefeld University, GERMANY

## Abstract

The growing multi-hazard environment to which millions of people in the world are exposed highlights the importance of making sure that populations are increasingly better prepared. The objective of this study was to report the levels of preparedness of a community exposed to two natural hazards and identify the primary sociodemographic characteristics of groups with different preparedness levels. A survey was conducted on 476 participants from two localities of the Atacama Region in the north of Chile during the spring of 2015. Their level of preparedness at home and work was assessed to face two types of natural hazards: earthquakes and floods.The findings show that participants are significantly better prepared to face earthquakes than floods, which sends a serious warning to local authorities, given that floods have caused the greatest human and material losses in the region’s recent history of natural disasters. Men claimed to be more prepared than women to face floods, something that the authors attribute to the particular characteristics of the main employment sectors for men and women in the region. The potential contribution of large companies on preparedness levels of communities in the areas in which they operate is discussed. The sociodemographic profile of individuals with the highest levels of preparedness in an environment with multiple natural hazards are people between 30 and 59 years of age, living with their partner and school-age children. The implications of the results pertaining to institutions responsible for developing disaster risk reduction plans, policies and programs in a multi-hazard environment are discussed.

## Introduction

A World Bank report that assessed the main natural disaster hotspots in the world [1] found that approximately 3.8 million km^2^ and 790 million individuals are exposed to at least two natural hazards, while 0.5 million km^2^ and 105 million individuals are exposed to three or more natural hazards. An increase in the magnitude, frequency and geographic distribution of natural disasters has been recently demonstrated, particularly for those related to climate change [2]. Records show that between 1994 and 2013, floods were the most frequent event (43% of all events registered), affecting approximately 2.5 billion people [3] and caused the greatest material costs and losses. In the same period, earthquakes and tsunamis caused the highest number of fatalities, estimated at around 750,000, with tsunamis being twenty times more lethal than earthquakes [3]. These statistics demonstrate the critical multi-hazard environment to which the global population is exposed.

The combination of human and economic losses, together with reconstruction costs, makes natural disasters both a humanitarian and an economic problem [1]. Between 1994 and 2013, natural disasters produced economic losses of more than USD 2.6 trillion [3]. More recently, in 2017, USD 314 billion were spent globally on damage related to natural disasters [4]. There is currently an unresolved debate regarding whether natural disasters hinder a country’s economic growth, given that the empirical evidence is somewhat heterogeneous [5]. However, high expenditure associated with natural disasters may reduce investment in other priority areas for a country, such as education, health, transport and security [5].

There are no countries or communities that are currently immune to the impact of natural disasters. It is, however, possible to reduce the effects of these events through management strategies focused on risk reduction [6]. Citizen preparedness strategies play a key role in reducing the effects of hazards that cannot be mitigated [6–8], as such strategies seek to improve the ability of individuals and communities to respond in the event of a natural disaster [7].

Chile, located in the Pacific Ring of Fire, is one of the countries that is most exposed to earthquakes/tsunamis and volcanic eruptions on the planet. Among the OECD member countries, Chile is the most exposed to natural hazards, where 54% of its population and 12.9% of its total surface area are exposed to three or more hazards [1]. Between 2008 and 2018, Chile was affected by ten natural disasters (earthquakes, tsunamis, wildfires, floods and volcanic eruptions), which translated into more than four million affected individuals and close to 800 fatalities [9]. The 2010 earthquake and tsunami alone caused the death of 562 people, and gave rise to more than USD 30 billion in material losses [10]. As such, the multi-hazard environment to which the population is exposed, and the high expenditure associated with natural disasters in Chile, emphasize the importance of adopting a multi-hazard approach to progress in the design of preparedness strategies. In order to move forward in this direction, the main objective of this study is to understand the current levels of preparedness of a community exposed to multiple natural hazards and identify the primary sociodemographic characteristics of groups that show different levels of preparedness. The results of this study are expected to contribute to the development of disaster risk reduction strategies and programs in multi-hazard environments.

### Preparedness in a multi-hazard environment

The complexity of territories and social structures expose communities to various hazards, both natural and man-made. Against this backdrop, the leading institutions responsible for disaster risk reduction worldwide indicate the importance of nations being able to assess, recognize and integrate the various hazards in their territories in their planning, in order to prepare the population to effectively mitigate the damages associated with these multiple hazards [11].

Although addressing a multi-hazard environment requires significant economic and political efforts, several studies have indicated that the multi-hazard approach has major benefits for the design of effective disaster risk reduction policies [12, 13]. A multi-hazard assessment permits not only more reliable territorial planning for a country’s inhabitants but also lets stakeholders show that focusing mitigation measures on a single hazard may increase vulnerability to others [12].

The main recommendations for multi-hazard environments include strengthening risk assessment within territories, informing the population of these risks to raise awareness, and establishing multi-disciplinary and multi-sectoral efforts to develop integrated public policies [14].

### Natural hazard preparedness

In recent decades, numerous studies have been focused on assessing individuals’ levels of preparedness for natural hazards, and the factors that promote the adoption of preparedness measures [15–17]. In the literature, there are different theoretical frameworks to conceptualize the adoption of preparedness measures to face natural hazards, where the Protective Action Decision Model [16, 18] and the Social-Cognitive Model [19, 20] are the most cited models. The first model recognizes that preparation is a behavior dependent on risk perception, previous experience and some demographic characteristics, among other variables. The social cognitive model focuses on the role of motivational factors on the decision to adopt preparedness actions, including awareness of the threat, anxiety, self-efficacy, and sense of community among others. Both models can help describe and understand the preparedness, however, for the purposes of the present study we incorporate elements of the Protective Action Decision Model, mainly in aspects related to the relation between sociodemographic factors and preparedness levels. This model also recognizes the role of experience that is relevant for this particular study considering that the communities that were studied had experienced both events.

One of the most common ways to study natural disaster preparedness levels is by characterizing these measures within the places where individuals spend most of their time, such as their homes (with their families) and their workplaces [21–23]. These areas are representative not only of the types of preparedness measures adopted by the population [22], but also the areas that people recognize as sources of common and relevant information for taking preparedness measures [24]. Preparedness actions involve developing plans, stockpiling of supplies and performing exercises and drills, all aimed to reduce the impact of the disaster [25]. These actions have been translated into recommendations, checklists and actions that organizations provide to households, communities and workplace in order to be prepared in case of a disaster. Response organizations recommend to frequently assess and evaluate whether these actions have been implemented.

Researchers have proposed several models to explain the decision to take action and implement preparedness actions, with a particular emphasis on the role that social cognitive processes [26]. Traditionally these models have emphasized the role of risk perception and have also shown that previous experience may be relevant, but with mixed results in relation with preparedness [18]. For the purposes of this study we focused on a community that had experienced different hazards in the past years, so we could examine also whether they appeared to be prepared to respond to different hazards.

#### Household preparedness

Researchers have mostly focused on understanding family preparedness when characterizing the preparedness levels of the population [23, 27]. Family preparedness has been researched and measured through different types of activities, such as survival measures, mitigation measures and planning measures [21, 23, 28–30]. Family planning measures in the face of natural hazards are those which are adopted least frequently, but whose importance is highly recognized among individuals [23, 30]. Family preparedness is recognized as the base from which other preparation actions take place [27].

#### Workplace preparedness

Despite the fact that research on natural disaster preparedness has primarily focused on family preparedness, the study of workplace preparedness is emerging as a relevant focus for research, given the role that organizations play in local economies, the lives of the people they employ and even recovery following natural disasters [31, 32].

As in the case of family preparedness, workplace preparedness involves planning activities, such as speaking with employees about the impact and importance of preparing the company for natural hazards, having an emergency plan in place, alternative energy supplies for the company’s operation following a natural disaster, insurance for this type of events, and the presence of an emergency kit in the company, among many others [21, 23, 27, 31, 33].

One factor that is most closely related to workplace preparedness is company size [27, 31, 33]. This is because companies with a larger number of employees have formalized risk reduction processes, and greater resources to implement them [31].

### Sociodemographic variables and preparedness level

Several of the studies that link *gender* to the adoption of preparedness measures conclude that women prepare more than men [29, 34], especially when it comes to measures related to creating a family emergency plan, the safety of household members, and the use of preparedness messages [35]. Similarly, it has been reported that *married people* or those who *live with their partner* show higher levels of preparedness than those who do not [23, 36, 37].

The *age* of subjects is also a predictor for the adoption of preparedness measures. While some studies conclude that older people adopt more preparedness measures, with one of the main reasons being previous exposure to and/or experience with natural disasters [29, 38]. In other studies researchers suggest that age is not significantly related to the adoption of preparedness measures [36, 39].

The *presence of children under 18 years of age in the household* is associated to higher levels of preparedness [37, 40, 41]. In a study conducted on a random sample of 1,158 households in Memphis, Tennessee, Edwards [39] suggests that parents feel responsible for the safety of children, and also because children receive more information (from their school environment) about how to prepare for natural hazards, motivating parents to implement these types of measures. Similarly, Pfefferbaum & North [42] indicate that parents are more concerned about what their children will experience during a natural disaster, which may prompt a desire to anticipate its consequences and to prepare in advance to mitigate any possible negative effects.

## Methodology

### Study area

**Fig 1:**
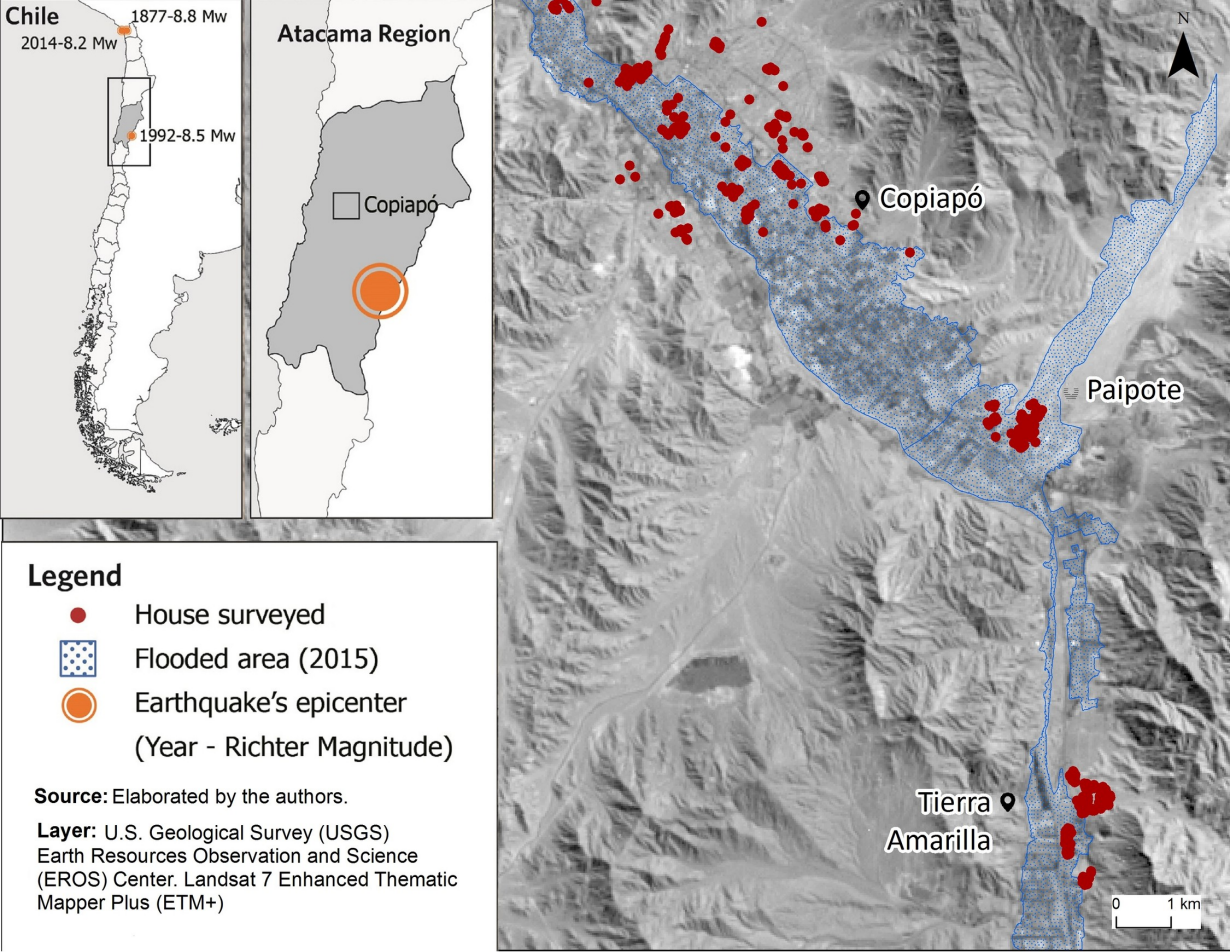
Map of the study area. The maps in the top left show the earthquakes that affected the Atacama Region. The map on the right shows the Copiapó and Tierra Amarilla municipalities, the flooded area of the 2015 event and the location of the households surveyed.

The research focused on the inhabitants of Copiapó and Tierra Amarilla municipalities (see Fig 1) in the Atacama Region in the north of Chile, since they are at risk of multiple natural hazards, particularly earthquakes and floods.

#### Geographic characteristics

The Atacama Region, Chile, has a surface area of 75,176 km^2^, equivalent to 9.94% of the country’s total (see Fig 1). Copiapó and Tierra Amarilla municipalities account for the 37% of the Region’s surface area. The climate of Copiapó and Tierra Amarilla is semi-arid, with scarce and light rainfall during the winter months. A phenomenon known as the “Altiplanic winter” takes place here, which triggers rainfall between the summer months of December and March [43]. The “Altiplanic winter” is the name given to the phenomenon of rainfall between December and March in the north of the country, as a result of moisture originating from the Atlantic Ocean [43]. However, rainfall has occurred during winter produced by the “Altiplanic winter” phenomenon that may intensify and produce extreme hydrometeorological events, due to the presence of weather patterns known as El Niño and La Niña [44].

#### Population

Copiapó and Tierra Amarilla municipalities (see Fig 1) are home to more than 60% of the Atacama Region’s population. The proportion of women in these municipalities is 48.6% and 42.4%, respectively [45]. Regarding age, the region’s population can be classified as follows: 19.3% are between 18 and 29 years of age, 21.0% are between 30 and 44 years of age, 19.3% are between 45 and 59 years of age, and 13.2% are above 60 years of age. A similar trend occurs for the populations of the Copiapó and Tierra Amarilla municipalities.

On December 2017, the unemployment rate in these localities reached 6.7%, slightly above the national average, which was 6.4% [46]. Mining is the sector which has the greatest influence on the country’s economic development, accounting for 10% of national GDP, generating 8.4% of national income, and representing at least half of total exports (55%) as of 2017 [47]. Currently, Chile is the largest copper producer in the world. As with other regions in the north of Chile, the main economic activity of Copiapó and Tierra Amarilla is mining (copper and other minerals), which accounts for 28% of the region’s GDP and is one of the main factors affecting employment rates. As of 2017, 15% of all workers in the region were employed in the mining sector, of which 92% were men [45].

#### Natural disasters in the study area

The localities of the Atacama Region have an extensive history of natural disasters, particularly extreme hydrometeorological events causing significant floods, with the events that took place in 1997 and 2015 considered the most catastrophic. In April 1997, intense rainfall caused rivers in the Atacama Region to overflow, producing floods that affected mostly to Copiapó (see Fig 1). A total of 22 people died, and material losses were estimated at USD 180 million [9]. Almost two decades later, in March 2015, there was a hydrometeorological event considered the largest in its history. More than 45mm of rain fell in approximately 48 hours [48]. The effects were devastating, mainly for the towns of Copiapó, Paipote, and Tierra Amarilla. A total of 31 people died, 16 were declared missing, 30,000 were displaced, and more than 164,000 people were affected by the event [49]. The material damages were estimated at more than USD 1.5 billion.

The Atacama Region’s localities are not only vulnerable to the occurrence of major floods but also, like the rest of the country, to severe geophysical events. Chile’s location in the Pacific Ring of Fire makes it one of the countries with the highest levels of seismic and volcanic activity on the planet. The largest earthquake recorded in the study area occurred in 1877, with a magnitude of 8.8 Mw on the Richter scale [50]. The second largest earthquake in the area occurred in 1922, with a magnitude of 8.5 Mw on the Richter scale [51]. The consequences of this event were devastating: 40% of houses were reduced to ruins, a further 45% requiring demolition, and the rest in dire need of repair [52]. The most recent earthquake in the area occurred in 2014 and is considered the third most destructive to hit the region. It had a magnitude of 8.2 Mw on the Richter scale, affected 13,000 homes, and caused the death of six people. Economic losses were estimated at more than USD 100 million. Despite these events, the scientific community has demonstrated that there are still subduction zones that have not been activated for more than 150 years, and as such the probability of another event with similar characteristics occurring in the near future is very high [53].

### Materials

The survey was separated into three sections, in which two types of natural hazard that affect the region were studied: earthquakes and floods. The first section contained questions about the level of preparedness for these two hazards. The second section assessed the participants’ prior experience of floods, and their evacuation experience in the latest event of 2015. Finally, the third section included questions about the participants’ sociodemographic characteristics. As this survey forms part of a larger study, only the measures that were used in this study are described below.

*Preparedness*. The earthquake and flood preparation scale was structured into two sub-scales; one to measure *household preparedness* (2 items) and another to measure *workplace preparedness* (3 items). The items on both sub-scales were adapted from previous studies [21, 23, 28, 29]. The participants were required to answer the questions associated with each sub-scale on each hazard (earthquake and flood) using a dichotomous scale (1) Yes, (0) No, as shown in Table 1. The set of preparedness actions of the questionnaire considered the main actions suggested by International Agencies as minimum elements of preparation of individuals. The yes/no answers to these questions would be indicative of participants' perception of preparedness rather than an objective measure of the actions they actually perform.

**Table 1:** Participant responses and reliability (alpha-Cronbach) for a) Earthquake preparation scale, and b) Flood preparation scale.

**a) Earthquake Preparation Scale**	**Yes (%)**	**No (%)**
***Workplace preparedness sub-scale (alpha = 0*.*86)***		
In your place of work, is there or do you have a plan regarding how to react in the event of an earthquake? (*n* = 177)	64.4	35.6
While you are working, do you know what the escape route is in the event of an earthquake? (*n* = 176)	72.7	27.3
Have there been drills for this earthquake plan in your workplace? (*n* = 175)	48.6	51.4
***Household preparedness sub-scale (alpha = 0*.*85)***		
At home, do you and your family have an action plan in the event of an earthquake? (*n* = 474)	74.3	25.7
At home, have you identified any escape routes in the event of an earthquake? (*n* = 473)	76.0	23.0
**b) Flood Preparation Scale**	**Yes (%)**	**No (%)**
***Workplace preparedness sub-scale (alpha = 0*.*89)***		
Thinking back on the flood of March 2015, was there or did you have an action plan for this event at your workplace? (*n* = 175)	24.6	75.4
Had there been drills for this plan at your workplace? (*n* = 173)	17.3	82.7
While you were working, did you know where the escape route was during the event of March 2015? (*n* = 174)	28.7	71.3
***Household preparedness sub-scale (alpha = 0*.*92)***		
For the flood of March 2015, had you identified the escape routes at home in the event of a major flood? (*n* = 472)	37.3	62.7
For the flood of March 2015, did you and your family have an action plan at home? (*n* = 472)	32.4	67.6

The differences in sample sizes are due to missing values. Each question is on a dichotomous scale (0)No; (1)Yes.

*Sociodemographic characteristics*. The participants were asked about various sociodemographic characteristics, including their age, gender, marital status, work activity, and whether children under 18 years of age live in their household.

### Procedure and participants

The understanding of the questionnaire was assessed and validated through a focus group directed by the research team. The sample was designed through simple random sampling, based on population forecasts for the Atacama Region developed by the National Statistics Institute of Chile in 2015. The first stage considered the random selection of geographic clusters (housing blocks) by block code. Then, households were selected using the Kish table and systematic sampling. Finally, people were selected on the basis of a quota system (to allow variability of gender and age). The survey took place between November and December 2015 with a statistically representative sample in the Copiapó and Tierra Amarilla municipalities. A group of interviewers contacted voluntary participants, who had to complete a paper questionnaire face to face at their homes (receiving no compensation of any form). Finally, a total of 476 people successfully completed the survey. The average age of the sample was 49 years (SD = 17.6 years, with a range of 18–94 years of age), and 66.9% of the participants were women. All procedures were approved by the Ethics Committee of the University Andres Bello.

Regarding participants’ work activity, 37.2% declared that they were employed, 35.5% were homemakers, 4.6% were studying, and 11.8% were retired. Of the total number of participants who declared that they were employed (179 participants), 45% were women. While the main employment sectors for women were services (social, personal and community) and commerce, for men, the main sectors were large and medium-scale mining, transport (mainly related to mining) and construction.

### Data analysis

First, a descriptive analysis of the data was carried out to assess the existence of coding errors and lost data. Then, an *internal consistency analysis* was performed on the full sample (*n = 476*). The internal consistency of each sub-scale was assessed through two measures: Cronbach's alpha and corrected item-total correlation. For the first measure, values above 0.7 suggest highly consistent scales [54]. For the second measure, values above 0.3 are suggested [55]. Item-total correlation values lower than the cutoff level imply that the item is not correlated with the sub-scale, and as such it should be omitted.

To characterize the profile of participants with higher (or lower) levels of preparedness, difference in means analyses (using post-hoc Tukey tests) and a Factorial ANOVA were carried out.

## Results

### Internal consistency

The internal consistency of the preparedness sub-scales was analyzed through alpha-Cronbach and corrected item-total correlation. For each participant, the preparedness sub-scales were calculated as the sum of the items that compose each one (see Table 1). For both hazards considered, the values of *household preparedness* range from 0 to 2, and for *workplace preparedness* range from 0 to 3. The sub-scales complied with all of the predefined requirements, and as such no items were eliminated. The *α*-Cronbach values for the household and workplace preparedness sub-scales for earthquakes and floods were above 0.8, and can be considered to be highly consistent (see Table 1).

### Earthquake vs. flood preparedness

Table 1 shows the descriptive analysis of the participants’ responses to earthquake and flood preparedness questions. Significant differences are observed when comparing the participants’ degree of *household preparedness* and *workplace preparedness* to face both hazards. While the majority of participants said that they were prepared for an earthquake both at work and at home (see Table 1A), a significantly lower proportion claimed to be prepared at work and at home for a flood (see Table 1B).

### Household preparedness

Table 2 shows the average values associated with *household preparedness* for earthquakes and floods, broken down by the sociodemographic characteristics of the sample. It can be observed that the participants stated that they were significantly more prepared at home for an earthquake than a flood (*p* < 0.001), regardless of their age, gender, marital status, and work activity. This result is an important warning sign for local and regulatory authorities, given that the recent history of natural disasters in the region reveals that floods have caused the greatest human and material losses.

**Table 2:** Mean values for earthquake and flood household preparedness*.

			Earthquake		Flood		p-value**
		%	Mean	*SD*	Mean	*SD*	
*All sample* (*n* = 476)		100.0	1.51	*0*.*80*	0.69	*0*.*92*	<0.001
*Gender*	Male	32.6	1.54^a^	*0*.*78*	0.82^a^	*0*.*95*	<0.001
	Female	67.4	1.50^a^	*0*.*82*	0.63^b^	*0*.*90*	<0.001
*Age Group*	18–29	18.7	1.37^a^	*0*.*88*	0.65^ab^	*0*.*90*	<0.001
	30–44	21.0	1.62^ab^	*0*.*70*	0.75^ab^	*0*.*93*	<0.001
	45–59	28.6	1.66^b^	*0*.*72*	0.85^a^	*0*.*95*	<0.001
	> 60	31.7	1.39^a^	*0*.*87*	0.55^b^	*0*.*87*	<0.001
*Marital Status*	Single-Separated-Divorced-Widowed	48.8	1.39^a^	*0*.*86*	0.59^a^	*0*.*88*	<0.001
	Married-Partner	51.2	1.63^b^	*0*.*73*	0.79^b^	*0*.*94*	<0.001
*School-age children*	At least one	55.0	1.55^a^	*0*.*79*	0.75^a^	*0*.*93*	<0.001
	None	45.0	1.47^a^	*0*.*82*	0.63^a^	*0*.*90*	<0.001
*Work Activity*	Working	37.2	1.57^a^	*0*.*76*	0.76^a^	*0*.*93*	<0.001
	Homemaker	35.5	1.48^a^	*0*.*83*	0.68^a^	*0*.*92*	<0.001
	Student	4.6	1.64^a^	*0*.*73*	0.77^a^	*0*.*97*	<0.001
	Retired	11.8	1.32^a^	*0*.*92*	0.47^a^	*0*.*84*	<0.001
	Other	10.9	1.56^a^	*0*.*75*	0.71^a^	*0*.*89*	<0.001

*Household preparedness sub-scale from [0–2]. Reading by column, mean values with different letters are significantly different at the *p < 0*.*10* level (Tukey’s HSD).

**Statistical significance between the mean difference for earthquake household preparedness and flood household preparedness.

Similarly, for both earthquakes and floods, it can be observed that the level of *household preparedness* by marital status and age group showed statistically significant differences (*p* < 0.1). In the former case, participants who were married or living with their partner declared higher levels of *household preparedness* than single, separated or widowed participants. In the latter case, subjects 60 years of age and above declared the lowest levels of *household preparedness* among the different age groups. In general, subjects between 30 and 59 years of age declared the highest levels of *household preparedness* to face both earthquakes and floods.

In the case of *household preparedness* for *floods*, women declared a lower level of preparedness compared to men.

To characterize the sociodemographic profile of subjects with higher (or lower) levels of declared *household preparedness*, a factorial ANOVA was carried out using sociodemographic characteristics as independent variables, and *household preparedness* as the dependent variable. The first columns in Table 3 show the results of the model for *household preparedness* for *earthquakes* (*F* = 204.292, *p* = 0.000), which explained 23.2% of the variance. The results suggest that the groups defined for the Work Activity variable have significantly different levels of *household preparedness* (*p* < 0.10). Similarly, the effects of two-way interactions (AgeGroup x MaritalStatus) and (WorkActivity x MaritalStatus) also showed significantly different levels of *household preparedness* for *earthquakes*. Three-way interactions (AgeGroup x MaritalStatus x Gender) and (WorkActivity x MaritalStatus x ChildrenAge) were statistically significant for *household preparedness* for *earthquakes*.

**Table 3:** Factorial ANOVA using sociodemographic variables as independent variables and earthquake (and flood) household preparedness as dependent variables.

Independent Variables	*Household Preparedness*					
	*Earthquakes*			*Floods*		
	MS	*F*	*p-value*	MS	*F*	*p-value*
Intercept	120.735	204.292	0.000	31.396	39.125	<0.001
Gender (G)	1.054	1.784	0.182	**4.684**	**5.837**	**0.016**
Age Group (A)	0.839	1.419	0.237	0.970	1.209	0.306
Children (C)	0.250	0.423	0.516	0.002	0.002	0.962
Marital Status (MS)	1.382	2.338	0.127	0.966	1.204	0.273
Work Activity (W)	**1.324**	**2.240**	**0.064**	1.415	1.764	0.135
A x MS	**2.037**	**3.447**	**0.017**	1.436	1.790	0.149
C x MS	0.113	0.191	0.662	0.698	0.870	0.351
G x MS	1.425	2.412	0.121	1.252	1.560	0.212
MS x W	**2.023**	**3.422**	**0.009**	0.247	0.308	0.873
A x C	1.143	1.934	0.123	1.620	2.019	0.111
G x A	0.686	1.161	0.324	1.564	1.948	0.121
A x W	0.922	1.561	0.146	1.228	1.530	0.156
G x C	0.065	0.110	0.740	0.425	0.529	0.467
C x W	0.636	1.076	0.368	0.805	1.003	0.406
G x W	0.026	0.044	0.996	0.825	1.028	0.393
A x C x MS	1.151	1.947	0.121	1.001	1.247	0.292
G x A x MS	**1.624**	**2.747**	**0.043**	0.738	0.919	0.431
A x MS x W	0.538	0.910	0.488	0.860	1.072	0.379
G x C x MS	0.174	0.294	0.588	0.071	0.089	0.766
C x MS x W	**1.982**	**3.354**	**0.010**	**3.862**	**4.813**	**0.001**
G x MS x W	1.289	2.181	0.114	0.142	0.177	0.838
G x A x C	1.111	1.880	0.132	0.752	0.937	0.423
A x C x W	0.896	1.516	0.171	**1.711**	**2.132**	**0.049**
G x A x W	0.038	0.065	0.978	0.964	1.201	0.309
G x C x W	0.934	1.581	0.193	1.367	1.703	0.166
Error	0.591			0.802		
*R*^*2*^	0.232			0.196		

**Fig 2:**
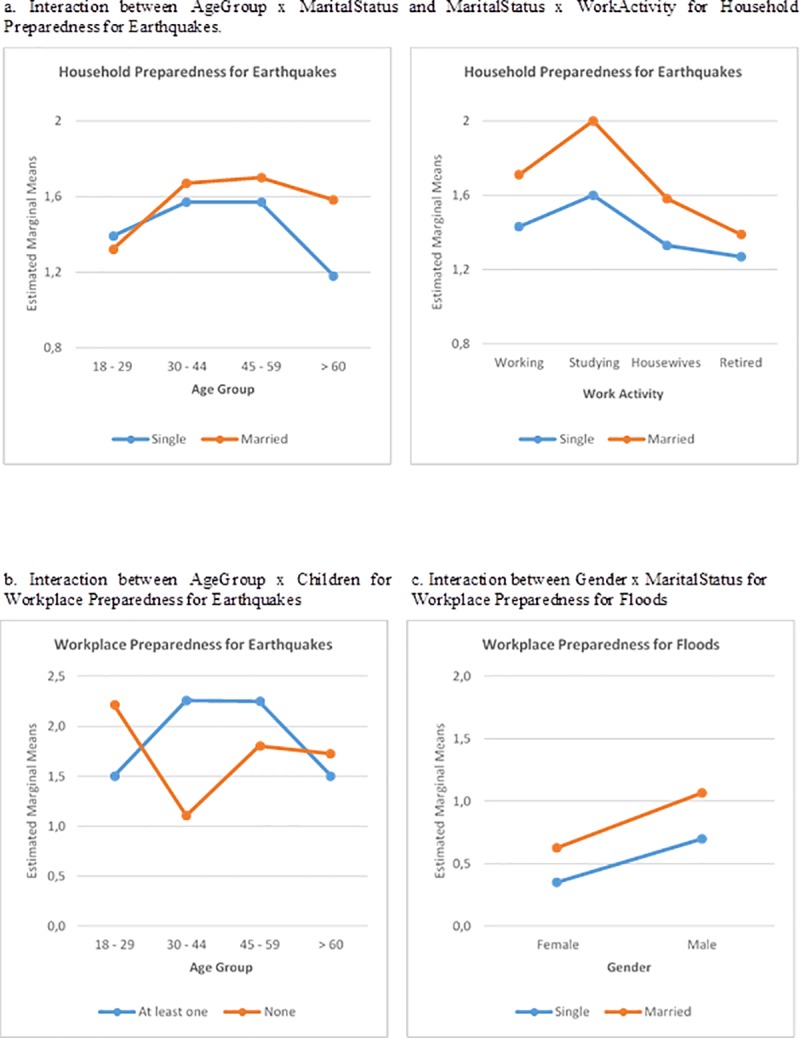
Interaction plots for household and workplace preparedness for earthquakes and floods.

Fig 2A. shows the groups associated with the two-way interaction between (AgeGroup x MaritalStatus) and (WorkActivity x MaritalStatus). Based on Table 2 and Fig 2A., it can be concluded that the profile of subjects with the highest level of *household preparedness* for *earthquakes* are between 30 and 59 years of age, married or living with their partner, and working or studying. On the other hand, the subjects with the lowest levels of *household preparedness* for *earthquakes* are those below 30 years old or above 60 years old, retired and single, separated or widowed. With regard to the three-way interactions, no clear trends were observed that enable to infer an evident profile.

The columns on the right-hand side of Table 3 show the results of the model for *household preparedness* for *floods* (*F* = 39.125, *p* = 0.000), which explained 19.6% of the variance. The only groups which show significantly different levels of *household preparedness* for *floods* were those defined by the Gender variable. Meanwhile, the three-way interactions (ChildrenAge x MaritalStatus x WorkActivity) and (ChildrenAge x AgeGroup x WorkActivity) were statistically significant for *household preparedness* for *floods*.

Based on the results shown in Table 2 and Table 3, we can conclude that men aged between 45 and 59 years of age who live with their partner declared the highest level of *household preparedness* for *floods*. On the other hand, the subjects who declared the lowest level of preparedness are women above 60 years of age who are single, separated, divorced or widowed. About the three-way interactions, no clear trends that suggest an evident profile may be inferred.

### Workplace preparedness

Table 4 shows the average values associated with *workplace preparedness* for earthquakes and floods, according to the sociodemographic characteristics of the sample (*n* = 179 participants who declared that they were employed). The results indicate that participants are significantly better prepared at work to face an earthquake than a flood (*p* < 0.001), regardless of their age, gender, and marital status.

**Table 4:** Mean values for earthquake and flood preparedness at work*.

			Earthquake		Flood		*p*-value**
		%	Mean	*SD*	Mean	*SD*	
*Sub-sample used* (*n* = 179)		100.0	1.87	*1*.*26*	0.71	*1*.*16*	<0.001
*Gender*	Male	55.9	1.76^a^	*1*.*28*	0.87^a^	*1*.*27*	<0.001
	Female	44.1	2.01^a^	*1*.*24*	0.51^b^	*0*.*95*	<0.001
*Age Group*	18–29	20.3	1.78^a^	*1*.*24*	0.66^a^	*1*.*06*	<0.001
	30–44	31.1	1.85^a^	*1*.*31*	0.75^a^	*1*.*27*	<0.001
	45–59	32.8	2.05^a^	*1*.*23*	0.86^a^	*1*.*23*	<0.001
	> 60	15.8	1.64^a^	*1*.*28*	0.39^a^	*0*.*83*	<0.001
*Marital Status*	Single-Separated-Divorced-Widow(er)	49.4	1.62^a^	*1*.*27*	0.56^a^	*1*.*06*	<0.001
	Married-Partner	50.6	2.11^b^	*1*.*22*	0.86^b^	*1*.*24*	<0.001
*School-age children*	At least one	55.9	2.01^a^	*1*.*23*	0.70^a^	*1*.*20*	<0.001
	None	44.1	1.68^b^	*1*.*29*	0.72^a^	*1*.*10*	<0.001

*Workplace preparedness sub-scale from [0–3]. Reading by column, mean values with different letters are significantly different at the *p < 0*.*10* level (Tukey’s HSD).

**Statistical significance between the mean difference for earthquake and flood workplace preparedness.

Both for earthquakes and floods, the MaritalStatus variable showed statistically significant differences (*p* < 0.10); that is, participants who are married or living with their partner declared higher levels of *workplace preparedness*.

In the case of *workplace preparedness* for *earthquakes*, participants who declared that they live with children under 18 years of age in their household showed higher levels of preparedness. Similar to the situation that occurred for *household preparedness*, women declared a lower level of *workplace preparedness* for *floods* compared to men.

The first columns of Table 5 show the results of the factorial ANOVA model using sociodemographic characteristics as independent variables and *workplace preparedness* for *earthquakes* as the dependent variable. The model explained 23.9% of the variance (*F* = 171.612, *p* = 0.000). The results indicate that the effects of the two-way interactions between the AgeGroup and Children variables show significantly different levels of *workplace preparedness* for *earthquakes*.

**Table 5:** Factorial ANOVA using sociodemographic variables as independent variables and earthquake (and flood) workplace preparedness as dependent variables.

Independent Variables	*Workplace Preparedness*					
	*Earthquakes*			*Floods*		
	MS	*F*	*p-value*	MS	*F*	*p-value*
Intercept	250.245	171.612	0.000	42.309	32.020	<0.001
Gender (G)	1.247	0.855	0.357	**6.491**	**4.913**	**0.028**
Age Group (A)	1.571	1.077	0.361	1.829	1.384	0.250
Children (C)	0.154	0.105	0.746	3.250	2.460	0.119
Marital Status (MS)	3.467	2.377	0.125	**8.078**	**6.114**	**0.015**
A x MS	1.047	0.718	0.543	0.826	0.625	0.600
C x MS	0.195	0.133	0.715	0.901	0.682	0.410
G x MS	0.003	0.002	0.965	**3.683**	**2.787**	**0.097**
A x C	**4.508**	**3.092**	**0.029**	1.112	0.841	0.473
G x A	2.615	1.793	0.151	1.286	0.973	0.407
G x C	0.333	0.228	0.634	2.091	1.583	0.210
A x C x MS	2.075	1.423	0.239	2.739	2.073	0.106
G x A x MS	0.570	0.391	0.760	0.753	0.570	0.636
G x C x MS	0.063	0.043	0.835	0.263	0.199	0.656
G x A x C	0.275	0.189	0.904	0.007	0.005	0.999
Error	1.458			1.321		
*R*^*2*^	0.239			0.177		

Fig 2B. shows the two-way interaction between the AgeGroup and Children variables. Based on the results shown in Table 5 and Fig 2B., it can be concluded that the profile of subjects who have the highest level of *workplace preparedness* for *earthquakes* are married or living with their partners, between 45 and 59 years of age, and have school-age children in their household. On the other hand, the participants with the lowest levels of *workplace preparedness* for earthquakes are those who are single (separated, divorced or widowed), above 60 years of age, and do not have school-age children living in the household.

The columns on the right-hand side of Table 5 show the results of the model using *workplace preparedness* for *floods* as the dependent variable. This model explained 17.7% of the variance (*F* = 32.020, *p* = 0.000). The results show that the groups defined by the Gender and MaritalStatus variables have significantly different levels of *workplace preparedness* (*p* < 0.10). Likewise, the two-way interaction effects of the Gender and MaritalStatus variables show significantly different levels of *workplace preparedness* for *floods*. Fig 2C. shows the two-way interaction between the Gender and MaritalStatus variables. Based on the results shown in Table 4 and Fig 2C., it may be concluded that while the profile of subjects with the highest declared level of *workplace preparedness* for *floods* is men who are married or living with their partner, the profile of those with the lowest level is women who are single, separated, divorced or widowed.

## Discussion

The objective of this study was to assess the level of *household* and *workplace preparedness* of people living in an area exposed to multiple natural hazards and identify those groups of people with different preparedness levels.

### Household and workplace preparedness

We conclude that significant differences exist in the preparedness levels declared by participants depending on the type of hazard analyzed. In fact, participants declared that they were significantly more prepared (both at home and at work) to face an earthquake than a flood, regardless of their age, gender, marital status and work activity. These results are an important warning sign for regulators and authorities, given that the recent history of natural disasters in the study area reveals that floods have caused the greatest human and material losses. Additionally, the influence of climate change is expected to produce an increase in weather phenomena, which would increase the frequency of extreme hydrometeorological events in the northern of Chile.

Among the reasons that may explain the above results is the fact that, historically, the country and the study area have placed greater emphasis on preparedness measures for earthquakes than for floods. In recent years, Chile has been affected by major earthquakes, with one of the most destructive one taking place on February 27, 2010 in the south of the country. This event caused great alarm and concern among citizens and government authorities, not only due to the destructive effects of the event, but also the shortcomings uncovered regarding the level of preparedness and coordination of government institutions responsible for disaster risk reduction. This situation received widespread media coverage, and was the subject of intense political debate which lasted for several years [56, 57].

In addition to the above, the scientific community has indicated that the recent earthquakes that have occurred in the north of the country provide evidence that there are still subduction zones which have not been activated in almost 150 years [53]. As such, the scientific community and authorities still expect a mega-earthquake to affect the study area. This situation has led to the implementation of many communication and community preparedness plans and programs to face a potential mega-earthquake in the region in recent decades. Awareness from communities about the likelihood of an earthquake is high and motivate them to be prepared for a future event.

Our results also show high levels of declared *workplace preparedness* for *earthquakes*, which could have its roots in the presence of large mining companies in the region. In fact, the mining industry has for decades constituted the main source of development in the region, in which large mining companies have played an important role in local economies. The presence of large mining companies represents one of the greatest opportunities for the development and implementation of preparedness programs in the face of hazards, given that, as they have large numbers of employees, their emergency risk reduction and response processes are more formalized.

Although the history of earthquakes in Chile have led both public and private-sector organizations to develop increasingly effective citizen and institutional preparedness strategies, the floods that occurred in 2015 demonstrated that the Atacama Region also reveal the need to improve preparedness strategies, programs and plans to face extreme hydrometeorological events. It is therefore recommended that institutions responsible for disaster risk reduction in the region design preparedness plans and programs that recognize and integrate the different hazards present in the region, given that the prioritization of preparedness strategies for one hazard may increase vulnerability to others.

### A sociodemographic profile of preparedness

Regarding the sociodemographic variables which are related to the *family* and *workplace preparedness* and in line with previous studies [29, 38], it is concluded that the subject’s age is significantly related to their declared levels of preparedness: in general, subjects of 30 to 59 years of age declared the highest levels of preparedness. Some authors posit that this could be explained because adults in this stage of life acquire greater care responsibilities (either for others or their own assets), which may give rise to increased interest in involving themselves in preparedness measures [41]. On the other hand, the low levels of preparedness declared by young people may be explained by the fact that, in general, they have a lower perception of natural disaster risk, which translates into lower willingness to adopt preparedness measures [58].

Being married or living with a partner was significantly related to higher levels of preparedness within the household. Previous studies have concluded that the presence of a significant other generates greater concern among subjects, and therefore greater willingness to prepare for potential natural disasters [39]. Regarding these arguments, the presence of school-age children in the household also produces higher levels of preparedness for natural hazards. Previous studies have argued that the presence of children in the household increases participation in preparedness measures due to the fact that children motivate the actions of adults, bring information regarding safety home from school, and because adults aim to protect children through this type of measures [39].

Finally, our results suggest that the level of preparedness for floods significantly differs depending on the subject’s gender: in general, men declare that they are more prepared for floods than women, contrary to what was expected. The authors attribute this result to the fact that the majority of men in the sample who are employed work in the large and medium-scale mining sector, while almost all women work in the services and commerce sectors. As mentioned throughout this study, the mining sector is the main source of employment and development in the region, characterized by the presence of large mining companies who provide direct employment to more than 15% of workers in the region, 92% of which are men [46]. Due to regulatory requirements, these companies have advanced security, hygiene and prevention standards which are frequently monitored. In line with previous studies [27, 31, 33], the employees of these large companies have greater learning and training opportunities with regard to emergency risk reduction and response processes, so it is reasonable to believe that those who work in such companies (mainly men) would have higher levels of preparedness for earthquakes and floods.

The above highlights the potential importance of large companies in the areas where they operate, not only because of their impact on local economies, but also due to their potential influence on communities’ degree of preparedness for natural disasters. Therefore, the presence of large companies in the region is a relevant and important factor to be considered by government authorities when designing disaster risk reduction programs. Families with some members working in large mining companies may improve their levels of family preparedness for natural disasters to the extent in which these members bring information and experience from work regarding emergency risk reduction and response processes home with them.

Based on the results obtained, we conclude that sociodemographic variables such as age, marital status, gender and the presence of school-age children in the household characterize the profile of subjects with greater (or lower) levels of *family* and *workplace preparedness* to face potential natural disasters in multi-hazard environments. One of the greatest influencers on the motivation to prepare for natural disasters is the presence of significant others in the household. In general, adults between 30 to 59 years of age who live with their partners and have school-age children in the household constitute the sociodemographic profile of subjects with the highest declared levels of preparedness to face potential natural disasters. On the other hand, adults below 30 years of age or above 60 years old who are single, separated or widowed, and do not have school-age children living in the household represent the profile of subjects with the lowest declared levels of preparedness to face a potential natural disaster. Groups that are less prepared should be target of interventions in order to raise awareness and motivate them to adopt preparedness actions.

Also, our findings reveal the need to continue investigating how people perceive/adopt the recommendations provided by local authorities (i.e., if they understand them and if they are capable of carrying them out), so to be able to evaluate which factors facilitate (or discourage) the adoption of preparedness actions. As some studies indicate, the preparedness actions are not always carried out by the individuals in the same way that authorities recommended it [59]. Therefore, it is necessary to keep a continuous dialogue between authorities and the civil population to effectively communicate preparedness strategies. This is a crucial element to go forward in the design of public policies that take into account the social, cultural and political context in which people live.

Finally, the institutions responsible for developing local disaster risk reduction plans and programs must appropriately characterize their target audiences if they expect to obtain more effective and efficient results. We hope that the results and conclusions reported in this study become a useful input to achieve this.

## Limitations

There are certain limitations to this study. The number of participants in the study was small, as it was made up of a representative sample of solely the Copiapó and Tierra Amarilla municipalities in the Atacama Region. Therefore, studies must be carried out in other cities in the country in order to capture the different events that they experience, as well as geographic and cultural differences.

The level of preparedness was assessed for participants solely through a single measure and using the self-reporting method. Even though dichotomous questions assess the perceived level of preparedness and do not allow to evaluate their objective level (or if they comprehend the emergency plan of their workplace or city), these questions provide an estimate of the basic actions of preparedness recommended by leading International Agencies, which should be done by individuals to face natural hazards. Although this method is extensively used in the literature, it does limit greater understanding of preparedness behavior.

## Supporting information

S1 Dataset Data set used in the research. (XLSX)
